# Virus-Induced Gene Silencing in *Chrysanthemum seticuspe* Using the Tomato Aspermy Virus Vector

**DOI:** 10.3390/plants11030430

**Published:** 2022-02-04

**Authors:** Hirotomo Murai, Tomofumi Mochizuki

**Affiliations:** Graduate School of Life and Environmental Sciences, Osaka Prefecture University, 1-1, Gakuen-cho, Naka-ku, Sakai 599-8531, Japan; sxc02083@edu.osakafu-u.ac.jp

**Keywords:** chrysanthemum, virus-induced gene silencing, tomato aspermy virus

## Abstract

*Chrysanthemum* is one of the most economically important flowers globally due to its high ornamental value. In recent years, a large percentage of the *Chrysanthemum seticuspe* genome has been determined, making this species useful as a model chrysanthemum plant. To fully utilize the genome’s information, efficient and rapid gene functional analysis methods are needed. In this study, we optimized the tomato aspermy virus (TAV) vector for virus-induced gene silencing (VIGS) in *C. seticuspe*. Conventional plant virus inoculation methods, such as the mechanical inoculation of viral RNA transcripts and agroinoculation into leaves, did not achieve successful TAV infections in *C. seticuspe*, but vacuum infiltration into sprouts was successful without symptoms. The TAV vector harboring 100 nucleotides of the *phytoene desaturase* (*PDS*) gene caused photobleaching phenotypes and a reduction in *CsPDS* expression in *C. seticuspe*. To our knowledge, this is the first report of VIGS in chrysanthemums.

## 1. Introduction

Chrysanthemum belongs to the Asteraceae family, which is the largest family of angiosperms [1]. The cultivated chrysanthemum, *Chrysanthemum morifolium* Ramat, is one of the most economically important flowers globally due to its high ornamental value [2]. However, cultivated chrysanthemum has a large genome and high polyploidy [3,4]; therefore, the genetic transformation of chrysanthemum is difficult and inefficient compared with that of other major model plants. *Chrysanthemum seticuspe* (Maxim.) Hand.-Mazz. (hereafter referred to as *C. seticuspe*) is a wild diploid chrysanthemum closely related to the cultivated chrysanthemum. These properties are preferable for a model plant; therefore, 89–97% of *C. seticuspe* genomes have been determined by next-generation sequencing [5,6]. To fully utilize this genome information, efficient gene functional analysis methods are needed instead of laboriously inefficient conventional plant transformations.

As a useful tool for gene functional analysis, various virus-induced gene silencing (VIGS) vectors have been developed in recent years [7]. VIGS depends on post-transcriptional gene silencing (PTGS) machinery in a sequence-specific manner. Briefly, double-stranded RNAs (dsRNAs), such as replication intermediates of plant RNA viruses and highly structured genomic RNA, are processed by Dicer-like proteins (DCLs) and generate small interfering RNAs (siRNAs). An RNA-induced silencing complex (RISC) incorporating siRNAs cleaves complementary single-stranded RNA (ssRNA) [8]. When a plant virus vector with a plant gene sequence replicates in plant cells, the complementary plant mRNA is also degraded through the PTGS machinery. VIGS does not require transformations of target plants; therefore, VIGS has an advantage over other laborious conventional methods for gene functional analyses [9].

Approximately 20 viruses, such as tomato aspermy virus (TAV), cucumber mosaic virus (CMV), chrysanthemum virus B, tomato spotted wilt virus, and chrysanthemum stem necrosis virus, infect chrysanthemums [10,11,12]. Among these viruses, we previously developed a TAV VIGS vector based on the TAV ChJ strain isolated from chrysanthemums, which induced silencing in *N. benthamiana* [13]. TAV belongs to the *Cucumovirus* genus of the *Bromoviridae* family and has positive-sense ssRNA genomes [14]. Genomic RNA1, 2, and 3 act as mRNAs encoding 1a and 2a replicase proteins and movement proteins (MPs), respectively. Subgenomic RNA4A and RNA4 are synthesized from RNA2 and RNA3, respectively, and a multifunctional 2b protein and capsid protein (CP) are expressed from RNA4A and RNA4, respectively [14,15,16,17].

In this report, we aimed to optimize the TAV VIGS vector for *C. seticuspe* to establish an efficient VIGS vector system for chrysanthemums. Vacuum infiltration into sprouts was successful for the inoculation of the TAV VIGS vector in *C. seticuspe*, whereas the mechanical inoculation of viral RNA transcripts and agroinoculation into leaves did not work. Although efficient TAV VIGS in *N. benthamiana* was achieved by the attenuation of the viral suppressor of RNA silencing (VSR) activity of the 2b protein [13], we demonstrated that the TAV vector with the wild-type 2b protein harboring the partial *C. seticuspe phytoene desaturase* (*CsPDS*) sequence caused photobleaching phenotypes in *C. seticuspe*. The TAV vector will be a useful rapid functional genomics tool in chrysanthemums.

## 2. Materials and Methods

### 2.1. Plant Material

Gojo-0, which is a self-compatible *C. seticuspe* pure line, was used as the chrysanthemum [18]. Five- or six-leaf-stage *C. seticuspe* plants were used for conventional inoculation methods, such as the mechanical inoculation of TAV RNA transcripts or agroinoculation into leaves, and germinated seeds were used for sprout vacuum infiltration.

### 2.2. TAV Vector

The TAV ChJ strain, isolated from *Chrysanthemum* in Japan, was used (GenBank, accession numbers: LC634031, LC634032, and LC634033) [13]. pTOPOT1, pTOPOT2, and pTOPOT3, in which TAV RNA1, 2, and 3 are cloned downstream of a T7 RNA polymerase promoter, respectively, were used for the mechanical inoculation of TAV RNA transcripts [13]. pJL89T1, pJL89T2, and pJL89T3, in which TAV RNA1, 2, and 3 are cloned downstream of a cauliflower mosaic virus 35S promoter, respectively, were used for agroinoculation [13]. pJL89T2_2b__ΔC61_, pJL89T2_2b__ΔC23_, pJL89T2_2bR46C_, and pJL89T2_2bS4042A_ containing deletions in C-terminus of 2b gene (ΔC61 and ΔC23) or amino acid substitutions in 2b gene (R46C and S4042A) in pJL89T2 were used as TAV 2b mutants with decreased VSR activity for agroinoculation [13].

### 2.3. VIGS Constructs

Total RNA was isolated from *C. seticuspe* using RNAiso Plus (Takara, Shiga, Japan) according to the manufacturer’s instructions. cDNA was amplified using ReverTra Ace (Toyobo, Osaka, Japan) and an oligo (dT) 15 primer. The 100 bp gene fragment of *CsPDS* (accession: KC202430.1) was amplified from the synthesized cDNA using CsPDS-Fw (5′-CAC CAG TTC CCG CTA GCT CGT TCA-3′) and CsPDS-Rv (5′-GGC CGC AAG CAA GTC ATA ATC CTG-3′) and cloned into the pGEM-T Easy Vector (Promega, Madison, WI) to create pGEM-CsPDS. Then, 100 partial nucleotides (nt) of the *CsPDS* gene were amplified from pGEM-CsPDS with primers CsPDS100-Fw (5′-ACT ACG CGT CAC CAG TTC CCG CTA GCT CG-3′) with an *Mlu*I site and CsPDS100-Rv (5′-ATC ACG CGT TGT CAA GGT CTG GGC GTG GA-3′) with an *Mlu*I site and cloned into the *Mlu*I sites of TAV RNA3 vector (pJL89T3_CS_ [13]) to create pJL89T3_CsPDS100-S_ and pJL89T3_CsPDS100-AS_. pJL89T3_CsPDS100-S_ and pJL89T3_CsPDS100-AS_ were used as VIGS constructs for the *PDS* gene in *C. seticuspe*.

### 2.4. Virus Inoculation

For the inoculation of TAV transcripts, after the linearization of the plasmid containing TAV cDNA (pTOPOT1, pTOPOT2, and pTOPOT3) by *Sac*I, the 3′ overhang was converted to a blunt end using T4 DNA polymerase (Applied Biological Materials, British Columbia, Canada). Infectious viral RNAs were synthesized using the RiboMAX large-scale RNA production system (Promega). Synthesized RNAs mixed in 50 mM Na_2_HPO_4_, pH 9.2, were mechanically inoculated into two fully expanded leaves of five- or six-leaf-stage *C. seticuspe* plants.

The *Rhizobium radiobacter* strain EHA105 was used for agroinoculation. Agroinoculation into leaves was performed as described previously with little modification [13]. Bacterial cultures were resuspended to a cell concentration of OD600 = 1.5.

For sprout vacuum infiltration, bacterial cultures were resuspended in infiltration buffer (10 mM MgCl_2_, 10 mM MES pH 5.6 and 200 µM acetosyringone) to a cell concentration of OD600 = 1.5. Equal volumes of bacterial cultures for RNA1, RNA2, and RNA3 were mixed and incubated at room temperature for 3 h. *C. seticuspe* seeds were germinated on filter paper presoaked with sterile water in a Petri dish for four days. The germinated *C. seticuspe* seeds were submerged in the bacterial culture mixture with 0.5% Tween 20 and infiltrated twice using a vacuum pump under 8 hPa pressure for 5 min. Infiltrated sprouts were transplanted into pots and covered with a chamber for 3–4 days to maintain humid conditions. After 3–4 days, the chamber was removed, and the plants were maintained at 24 °C with a 16 h light/8 h dark cycle.

### 2.5. Detection of Viral CP by a Dot Immunobinding Assay (DIBA)

The leaf tissues were ground with a tenfold volume of phosphate-buffered saline (PBST) containing 0.05% Tween 20. The homogenates were spotted onto a BioTrace NT Membrane (Pall Corporation, Port Washington, NY, USA) and blocked with 3% skim milk-Tris-buffered saline (TTBS) containing 0.05% Tween 20. The membrane was washed for 10 min with PBST, and the primary antibody against TAV (1:5000 dilution in PBST) was added and incubated at 4 °C overnight. After being washed twice, the membrane was incubated with alkaline phosphatase-conjugated secondary antibody (1:10,000 dilution in 3% skim milk-TTBS) for 2 h at room temperature. Staining was performed using 5-bromo-4-chloro-3-indolyl phosphate and nitro blue tetrazolium.

### 2.6. Detection of Viral RNA by RT–PCR and Gene Expression Analysis by RT–qPCR

Total RNA was isolated from leaf tissues using ISOGEN II (Nippon Gene, Tokyo, Japan) according to the manufacturer’s instructions. The detection of viral RNA by RT–PCR was performed as described previously [13].

To analyze gene expression levels in plants showing silencing phenotypes, RT–qPCR was performed. Total RNA was treated with a gDNA Removal Kit (Jena Bioscience, Jena, Germany), and 250 ng of gDNA-free total RNA was used as a template. The RT reaction was performed using ReverTra Ace (Toyobo) with an oligo (dT) 15 primer. The resulting five-fold-diluted RT product was then used as a template for qPCR. Real-time PCR was performed using a KAPA SYBR FAST qPCR Master Mix kit (Kapa Biosystems, Wilmington, MA, USA) with QuantStudio 3 (Thermo Fisher Scientific, Waltham, MA, USA) according to the manufacturer’s instructions. For the analysis of *CsPDS* expression levels, the primer pair CsPDS-qFw (5′-TCC TGA AGA AAC CGG TTT AG-3′) and CsPDS-qRv (5′-CTG AGG TGT TTA TTG CCA TG-3′) was used. For the analysis of CsEF1α as an internal control, a primer pair (5′-CTT GTT GCT TGA TGA CTG TGG-3′) and (5′-ACC ATT CAA GCG ACA GAC TC-3′) was used [19]. A standard curve method based on the serial dilution of the TAV empty vector was used to calculate the relative expression level of each gene. Each RT–qPCR analysis was performed with five to six biological replicates and analyzed statistically by Student’s t-test.

## 3. Results

### 3.1. TAV Inoculation Method for C. seticuspe

**Figure 1:**
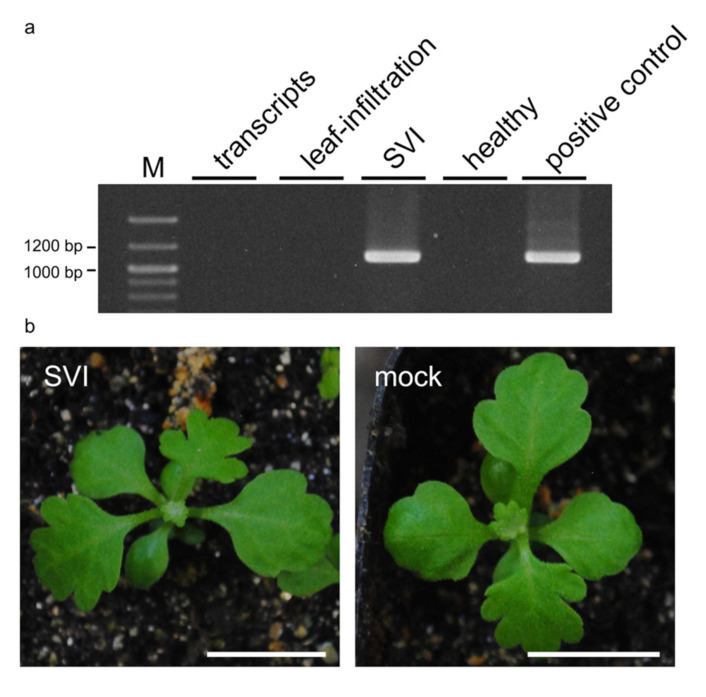
Efficient inoculation method of tomato aspermy virus (TAV) into *Chrysanthemum seticuspe*. (**a**) RT–PCR detection of TAV in *C. seticuspe* inoculated with the TAV cDNA clone. M, DNA size marker; the transcripts, leaf infiltration, and SVI indicate that RNA was extracted from the *C. seticuspe* inoculated by the mechanical inoculation of viral transcripts, agroinoculation into leaves, and vacuum infiltration into sprouts, respectively; healthy, healthy plant; positive control, RNA extracted from the *Nicotiana benthamiana* inoculated with TAV-ChJ. (**b**) *C. seticuspe* plants that the TAV was detected at 28 days postinoculation (dpi) (SVI). Mock plants were inoculated with a pJL89 binary vector by SVI. Bars, 10 mm.

We previously developed infectious clones of TAV-ChJ isolated from chrysanthemums and performed VIGS in *N. benthamiana* but not in *C. seticuspe* [13]. Therefore, we first confirmed the infectivity of TAV cDNA clones to *C. seticuspe*. The mechanical inoculation of TAV transcripts and the agroinoculation of TAV cDNA clones into leaves were not successful (Table 1), suggesting that these conventional inoculation methods are not suitable for *C. seticuspe*. It has been reported that the inoculation of strawberry vein banding virus (SVBV) by vacuum infiltration was more effective than that by agroinoculation using syringe infiltration [20]. Therefore, as an alternative TAV inoculation method, we performed sprout vacuum infiltration (SVI) [21]. *C. seticuspe* seeds were germinated in a Petri dish, and the germinated sprouts were vacuum-infiltrated with bacterial cultures containing TAV cDNA clones. TAV was detected in uninoculated upper leaves from ten out of twenty of the vacuum-infiltrated *C. seticuspe* by RT–PCR or DIBA at 28 days post-inoculation (dpi) (Table 1 and Figure 1a). Importantly, all TAV-detected plants showed no symptoms (Figure 1b). These results suggest that SVI is a suitable inoculation method for *C. seticuspe*.

### 3.2. Infectivity of 2b Mutants That Have Various VSR Activities to C. seticuspe

Our recent data suggested that efficient VIGS induction by the TAV-ChJ vector in *N. benthamiana* was achieved when the VSR activity of the 2b protein was attenuated [13]. Therefore, we examined the effect of 2b VSR activity on TAV infection of *C. seticuspe*. TAVs containing the ORF for the wild-type or mutated 2b protein retaining various VSR activities (ΔC61, ΔC23, R46C, and S4042A) were vacuum infiltrated into *C. seticuspe* sprouts. The deletion mutants ΔC61 and ΔC23 was not detected at 28 dpi (Table 2). S4042A and R46C mutants were detected with detection percentages of 4.2 and 15%, respectively (Table 2), which were drastically lower than that of the wild type (50%). These results suggest that efficient TAV infection of *C. seticuspe* requires intact VSR activity of the 2b protein. Considering the lower detection percentage of 2b mutants, we decided to use wild-type TAV for VIGS in *C. seticuspe*.

### 3.3. VIGS in C. seticuspe

**Figure 2:**
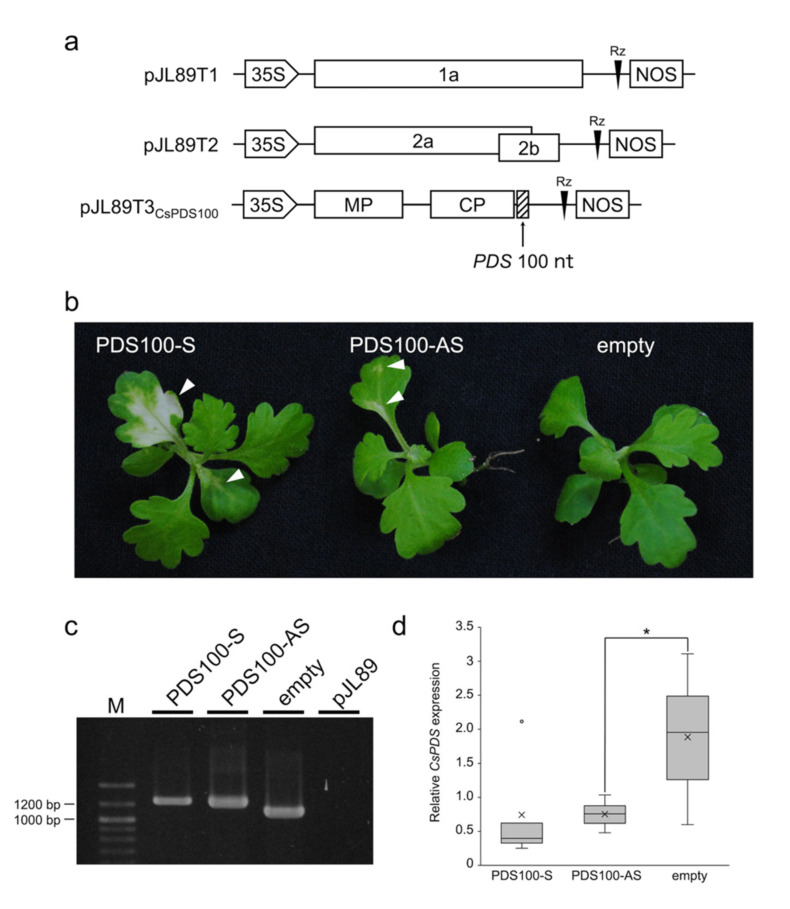
Virus-induced gene silencing (VIGS) induction in *Chrysanthemum seticuspe* by the tomato aspermy virus (TAV) vector. (**a**) Schematic representation of the VIGS constructs. The partial 100 nucleotides of the *C. seticuspe phytoene desaturase* (*PDS*) gene were introduced just downstream of the capsid protein (CP) open reading frame in TAV RNA3 (pJL89T3_CsPDS100_) and agroinoculated with pJL89T1 and pJL89T2. The TAV genomes were cloned between the 35S promoter (35S) and the nopaline synthase terminator (NOS). The ribozyme sequence of hepatitis delta virus ribozyme (Rz) is located just downstream of the 3′ untranslated region of TAV cDNA. (**b**) *C. seticuspe* infected with the TAV vector described in Panel A at 28 days postinoculation (dpi). The photobleaching areas are indicated by white arrowheads. (**c**) RT–PCR analysis of the TAV vector harboring the partial *CsPDS* sequence. RNA samples were extracted from *C. seticuspe* leaves at 28 dpi. pJL89 was used as a negative control. TAV not harboring the partial *CsPDS* sequence was used as the empty vector control. The RT–PCR product of the TAV vector harboring the partial *CsPDS* sequence was approximately 1200 base pairs (bp), whereas that of the TAV empty vector was approximately 1100 bp. (**d**) RT–qPCR analysis of *CsPDS* expression levels. RNA samples were extracted from *C. seticuspe* leaves at 28 dpi. TAV not harboring the partial *CsPDS* sequence was used as the empty vector control. Each RT–qPCR analysis was performed with five to six biological replicates. Boxes show the interquartile ranges including 25–75% of the values, and whiskers indicate the highest and lowest values of data. Horizontal lines and cross marks in boxes indicate the medians and means, respectively. The value outside 1.5 times the interquartile range between 25% and 75% of each group was considered an outlier and indicated with a dot. Asterisks indicate significant differences by Student’s t-test at *p* < 0.05.

The 100-nucleotide (nt) fragment from the *CsPDS* gene, a visible VIGS marker gene, was introduced downstream of the CP ORF in pJL89T3 in a sense (CsPDS100-S) or antisense orientation (CsPDS100-AS) and vacuum-infiltrated with pJL89T1 and pJL89T2 (Figure 2a). *C. seticuspe* plants inoculated with TAV-CsPDS100-S and TAV-CsPDS100-AS showed photobleaching phenotypes, whereas the TAV empty vector did not induce photobleaching phenotypes (Figure 2b). The silencing frequency was defined as the percent of plants showing silencing phenotypes after inoculation with VIGS vectors [22], and the silencing frequencies of TAV-CsPDS100-S and TAV-CsPDS100-AS were 48.7 ± 13.7% and 24.5 ± 10.9% (8–18 plants were inoculated with three replicates), respectively. The silencing frequency of photobleaching phenotypes induced by TAV-CsPDS100-S was nearly equal to the detection percentage of the TAV 2b wt (Table 2), suggesting that TAV-CsPDS100-S can induce photobleaching phenotypes at a high rate if they are infected. The photobleaching areas of TAV-CsPDS100-S were larger than those of TAV-CsPDS100-AS (Figure 2b). Consistent with the photobleaching phenotype, RT–qPCR showed that the relative *CsPDS* expression levels of TAV-CsPDS100-S and TAV-CsPDS100-AS were decreased compared with that of the TAV empty vector (Figure 2d). The presence of the TAV vector harboring the partial *CsPDS* sequence was confirmed by RT–PCR in the photobleached tissues of *C. seticuspe* (Figure 2c). These results indicate that the photobleaching phenotypes were induced by TAV vectors containing the partial *CsPDS* sequence, and VIGS was more effective when the partial *CsPDS* sequence was inserted in a sense orientation.

## 4. Discussion

To utilize most of the advantages of VIGS, the inoculation method of the VIGS vector should be efficient and simple. We first attempted conventional plant virus inoculation methods, such as mechanical inoculation and agroinoculation, but these two methods failed to establish TAV infections in *C. seticuspe* (Table 1 and Figure 1a). We showed that SVI achieved successful TAV infections in *C. seticuspe* when the same bacterial inoculums, except for the acetosyringone concentration (100 and 200 µM for leaf infiltration and SVI, respectively), were used. It has been reported that vacuum infiltration of the SVBV cDNA clone was more infectious than that delivered by agroinfiltration into leaves [20]. The reason why the difference in infectivity occurred between these two agroinfiltration methods is unclear, but there are two differences between leaf infiltration and SVI. First, *C. seticuspe* sprouts were used for SVI soon after germination, whereas five- or six-leaf-stage *C. seticuspe* were infiltrated in the leaf infiltration. Second, bacterial mixtures were vacuum-infiltrated in whole seedlings, including cotyledons, stems, and roots, for the SVI, whereas bacterial mixtures were infiltrated into only true leaves for leaf infiltration. Namely, the timing and sites of the agrobacterium introduction (and/or viral RNA, which is transcribed from T-DNA) were different between these two agroinfiltration methods. To achieve efficient TAV infections in *C. seticuspe*, it may be suitable to agroinoculate the whole seedlings when *C. seticuspe* is extremely young and more susceptible to viral infection. It has been reported that *C. seticuspe* were infected with chrysanthemum stunt viroid by the injection of viroid transcripts into stems [23], supporting the importance of the pathogen entering the site for successful infection.

The TAV 2b protein is a counterdefense protein against RNA silencing. Some mutations in TAV 2b decreased its VSR activity but did not cause a loss of infectivity of TAV in *N. benthamiana* [13]. However, in this study, the TAV 2b mutants lost or showed extremely decreased infectivity in *C. seticuspe* (Table 2), showing that the intact 2b protein is required for the efficient infection of TAV in *C. seticuspe*. Previously, we showed that decreased VSR activity of the 2b protein was required for VIGS of the TAV vector in *N. benthamiana* [13]. Recently, Zhou et al. reported that the percentage of silenced plants induced by the tobacco rattle virus (TRV) vector expressing the TAV 2b protein was only 3% in pepper, whereas that induced by the TRV vector expressing the CMV 2b protein was 93% [24]. Thus, TAV 2b seems to suppress VIGS. However, in this study, the TAV VIGS vector with wild-type 2b induced *PDS* silencing in *C. seticuspe* with a higher rate of 25–49% (Figure 2). Considering these reports and our results, it is suggested that the effect of TAV 2b VSR activity on VIGS is different between Asteraceae and Solanaceae plants or that the PTGS machinery itself is different between these two plant families. *N. benthamiana* has been used as a model plant for plant virology for a long time due to its high susceptibility to pathogens [25]. In addition, many RNA silencing-based crop protection systems have been reported in Solanaceae plants because this family includes economically important crops such as tomato and potato [26]. For these reasons, much information about the RNA silencing machinery is accumulated in Solanaceae plants. To our knowledge, the RNA silencing mechanism of Asteraceae has not been investigated. A detailed analysis of the RNA silencing mechanism of Asteraceae plants may help to provide a better understanding of the effect of TAV 2b VSR activity on VIGS.

The orientation of the target sequence affects VIGS effectiveness. Some reports showed that antisense orientation was more effective for VIGS [27,28,29], but others showed that there was no difference between the sense and antisense orientation [30,31,32,33,34]. These reports differ in the viral vectors used, the host plants, and the target genes; thus, the effect of gene orientation to induce VIGS may depend on the experimental conditions. In this study, the TAV vector harboring the partial *CsPDS* sequence in any orientation caused photobleaching phenotypes, but the antisense orientation did not induce more effective VIGS against *CsPDS* than the sense orientation (Figure 2b,d). It has been reported that the position or sequence of the target gene also affects VIGS [29,30,31]. Therefore, it remains possible that other positions or sequences of the *CsPDS* gene may result in a different VIGS effect in a sense and antisense orientation.

In summary, we optimized the inoculation method and VSR activity of the 2b protein for the TAV VIGS system in *C. seticuspe*. The optimized TAV VIGS vector system silenced a *C. seticuspe* endogenous gene, but the silenced phenotype was in a limited area. Therefore, modifications allowing VIGS in a broader area will provide more useful functional genomics tools in chrysanthemums.

## Figures and Tables

**Table 1:** Efficiency of different inoculation methods of tomato aspermy virus (TAV) in *Chrysanthemum seticuspe*.

Inoculation Method	No. of Inoculated *C. seticuspe* ^a^	No. of TAV-Detected *C. seticuspe* ^b^	Detection Percentage ^c^
Transcripts	12	0	0%
Leaf infiltration	12	0	0%
Sprout vacuum infiltration	20	10	50%

^a^ The number of *Chrysanthemum seticuspe* inoculated with TAV; ^b^ the number of *C. seticuspe* in which TAV was detected in the upper leaves by RT–PCR or DIBA; ^c^ percentage of TAV-detected plants/total number of inoculated plants.

**Table 2:** Infection of tomato aspermy virus (TAV) 2b mutants in *Chrysanthemum seticuspe*.

2b Variation	No. of Inoculated *C. seticuspe* ^a^	No. of TAV-Detected *C. seticuspe* ^b^	Detection Percentage ^c^
wt	20	10	50%
ΔC61	22	0	0%
ΔC23	19	0	0%
R46C	20	3	15%
S4042A	24	1	4.2%

^a^ The number of *Chrysanthemum seticuspe* inoculated with TAV; ^b^ the number of *C. seticuspe* in which TAV was detected in the upper leaves confirmed by RT–PCR or DIBA; ^c^ percentage of TAV-detected plants/total number of inoculated plants.

## Data Availability

Not applicable.
